# Leveraging Virtual Reality Technology in Saudi Arabia to Enhance Training: A New Paradigm for Developing Women Leaders in the Workplace

**DOI:** 10.7759/cureus.81769

**Published:** 2025-04-05

**Authors:** Latefa Hamad Al Fryan, Mahasin Ibrahim Shomo, Rawad Hammad, Mohamed Imam

**Affiliations:** 1 Department of Educational Technology, Princess Nourah Bint Abdulrahman University, Riyadh, SAU; 2 Department of Administrative Sciences, Princess Nourah Bint Abdulrahman University, Riyadh, SAU; 3 Department of Computer Science and Digital Technologies, University of East London, London, GBR; 4 Smart Health Centre, University of East London, London, GBR

**Keywords:** education, extended reality, leadership, management, virtual reality

## Abstract

Background: Gender bias and workplace challenges continue to hinder the career advancement of women in leadership roles. As Saudi Arabia aligns with Vision 2030, empowering Saudi women leaders (SWLs) through innovative training methods has become a national priority. Virtual reality (VR) technology offers immersive, interactive environments that simulate workplace challenges, fostering skill development and bridging the gap between theory and practice, particularly beneficial for women leaders who can practice navigating leadership scenarios safely.

Objective: This pilot study investigates the effectiveness of VR technology in enhancing the leadership competencies, digital communication skills, and workplace adaptability of SWLs.

Methods: A total of 52 SWLs from public and private organizations in Riyadh participated in a two-week VR-based training program focused on real-life leadership scenarios and workplace challenges. Designed by Princess Nourah University’s Women Leadership Center in collaboration with the Massachusetts Institute of Technology (MIT), which provided VR design expertise, the program addressed gender bias and leadership stereotypes. Data were collected using a System Usability Scale (SUS)-based questionnaire combined with leadership training evaluation items, which assessed both VR system usability and training effectiveness. Descriptive and inferential statistics (including Pearson correlation coefficients) were used to analyse the data.

Results: Participants reported high levels of satisfaction with the usability and immersive nature of VR training (mean scores ranging from 3.95 to 4.11 on a five-point scale). The VR system was rated as intuitive and engaging, with participants quickly adapting to its use (mean score: 4.11). The training facilitated interactive and stress-free learning experiences (mean score: 4.08), though some participants expressed uncertainty regarding applying VR skills to day-to-day leadership tasks such as conflict resolution and performance management (mean score: 3.05).

Conclusion: VR technology, as indicated by mean usability scores around 4.0 on a five-point scale, is a promising tool for developing leadership competencies among SWLs. While participants demonstrated strong engagement and skill acquisition, further refinement is needed to align training scenarios with real-world tasks. Expanding carefully evaluated VR-based training programs can advance women's leadership development and support Saudi Vision 2030 objectives.

## Introduction

Gender bias is a persistent obstacle for women pursuing leadership positions. Previous studies [1-3] reveal that gender bias and discrimination, including pay disparities and promotion barriers, are prevalent in workplaces, challenging efforts to foster diversity and inclusivity. Post COVID-19, women remain underrepresented in leadership roles across the USA despite low unemployment rates [2]. This underrepresentation also resonates globally, including in Saudi Arabia. Research indicates women often face biased evaluations, approximately 10-15% lower starting wages, and limited mentorship opportunities [4,5]. Furthermore, Heilman and Caleo [3] found that women often must exceed performance expectations for tasks perceived as "male-dominated", making leadership pursuits difficult.

Building on evidence of gender bias, positive workplace communication and inclusive environments are crucial for enabling women to reach their full potential. Due to systemic barriers, Campos et al. [6] argue that women frequently encounter limited career advancement opportunities, including a lack of promotions to leadership positions and exclusion from critical decision-making roles. In Saudi Arabia, female workforce participation rose 33% since 2017, yet Saudi women leaders (SWLs) require targeted training programs to enhance their leadership abilities, digital skills, and workplace communication. These efforts align with Saudi Vision 2030, which aims to empower women to comprise 30% of leadership roles by 2030.

Given the lack of empirical data on virtual reality (VR) training for female leadership in Saudi Arabia, this study investigates the efficacy of VR training in enhancing SWLs' leadership capacities. Similar to how VR has transformed medical training by reducing errors by 40% in surgical simulation [7], this technology provides immersive and safe learning environments for leadership development. While initial hardware setup can be costly, VR's long-term adaptability and affordability have enabled its effective use in construction, education, and healthcare [4]. In education, VR fosters interactive and engaging learning experiences, simplifying complex concepts and increasing knowledge retention by up to 30% compared to traditional methods [5].

SWLs, who currently form approximately 20% of mid-level managers, face challenges navigating mixed workplaces and labour market dynamics. Despite a 33% increase in female managers since 2017, gaps in leadership training persist, particularly regarding collaboration, communication, and digital skills development. The study addresses the need for immersive VR-based training, which differs from conventional workshops by providing experiential learning in simulated environments, to help SWLs overcome these challenges, improve workplace performance, and adopt effective leadership practices.

Research objectives

Based on the literature review and identified research gaps, this study aims to investigate the efficacy of VR technology as an innovative training method for developing leadership competencies among SWLs. The research is guided by the following specific objectives:

To evaluate the usability and effectiveness of VR systems in leadership training contexts, particularly examining how SWLs interact with and adapt to immersive learning environments.

To assess the impact of VR-based training on participants' professional performance, specifically their leadership competencies, digital communication skills, and ability to navigate complex workplace dynamics.

To explore SWLs' attitudes and perceptions toward the use of VR technology in human resource development initiatives, including their willingness to engage with similar technological interventions in future training programs.

Through these interconnected research objectives, the study seeks to contribute to the growing body of knowledge on innovative leadership development approaches and provide practical insights for organizations seeking to empower women leaders through technological interventions, thereby supporting Saudi Vision 2030's goals for women's advancement in leadership positions.

## Materials and methods

Study design

This pilot study utilized a mixed descriptive and correlational design to evaluate the effectiveness of VR training in enhancing the leadership competencies of SWLs. The study focused on (1) usability of the VR system, (2) participant engagement with the training content, and (3) perceived effectiveness of VR technology in addressing workplace challenges, specifically emphasizing leadership performance, digital communication skills, and the ability to navigate complex professional environments.

Research collaboration

This research represents a collaborative effort between Princess Nourah University (PNU) and the Massachusetts Institute of Technology (MIT), with additional support from researchers at other institutions. The collaboration was structured with clear role delineation: PNU's Women Leadership Center designed the study protocol and managed participant recruitment from Saudi organizations, while MIT researchers provided technical expertise in VR system design and implementation. Data collection was conducted on-site at PNU by local researchers, with weekly virtual coordination meetings held between all research team members. Data analysis was performed jointly, with MIT researchers focusing on system usability metrics and PNU researchers analysing leadership competency outcomes. The manuscript was prepared through a shared online document platform with each author contributing to their area of expertise and all authors reviewing the final manuscript. This collaborative approach ensured that both local cultural insights and international technical standards were incorporated throughout the research process.

Participants

The study included 52 SWLs from public and private organizations in Riyadh, Saudi Arabia, recruited via email invitations to organizations participating in leadership development programs. Participants were selected using convenience sampling based on their enrolment in a leadership training program conducted by the PNU Women Leadership Centre in collaboration with MIT. Inclusion criteria required active attendance in all VR training sessions. Exclusion criteria included incomplete participation, prior extensive VR experience, and inability to use VR headsets.

A diverse group of individuals with varied educational qualifications, professional experience, and organizational backgrounds was deliberately selected to ensure a representative sample. A total of 60 SWLs initially enrolled in the program; however, eight participants who failed to attend the VR training session were subsequently excluded, resulting in a final sample of 52 respondents.

Intervention

The VR training program, conducted over two weeks in March 2022 (six sessions, each approximately three hours in duration), was designed to address gender biases and leadership stereotypes through immersive and interactive virtual scenarios. Training sessions included a blend of (a) individual VR activities (approximately a day), where participants practised handling workplace challenges; (b) group discussions (90 minutes each) focused on leadership strategies; (c) interactive exercises (30-45 minutes each) emphasizing digital skill acquisition and communication.

The VR training was tailored to replicate real-world scenarios [6-9], allowing participants to engage in hands-on problem-solving and leadership exercises in a safe, controlled environment.

Study tool

The data were collected using a questionnaire based on the System Usability Scale (SUS), developed by John Brooke [10], a well-established tool for assessing usability. The SUS questionnaire was modified to evaluate the usability of VR systems and participants' training experiences across two key themes. The first theme, usability of the VR System (10 items), measured the ease of interaction and confidence in using the VR system. The second theme, evaluation of VR training (14 items), evaluated the impact of VR technology on leadership training, focusing on task performance, comfort, and overall experience.

The questionnaire utilized in this study consisted of two main sections, as detailed in the Appendix. Participants responded to the questionnaire using a five-point Likert scale, ranging from 1 (strongly disagree) to 5 (strongly agree). The questionnaire also collected demographic information, including educational qualifications, professional experience, and employment sector.

Validity and reliability

Subscale 1 (usability of the VR system) had a Cronbach's alpha of 0.843, while subscale 2 (evaluation of VR training) had a Cronbach's alpha of 0.897, indicating high internal consistency for both measurement dimensions. The results revealed significant correlations, ranging from 0.682 to 0.951 (p < 0.05), thereby establishing the structural validity of the tool.

Cronbach’s alpha was employed to establish reliability. The reliability values ranged from 0.843 to 0.897, indicating high internal consistency and confirming that the tool was appropriate for data collection.

Ethical considerations

This study was reviewed and approved by the Institutional Review Board of Princess Nourah University (Approval Number: HAP-01-R-059) on May 10, 2022. All participants provided written informed consent prior to engaging in the VR training program.

Participants did not receive monetary compensation for their participation; however, they received a certificate of completion as part of their professional development.

Statistical analysis

Data were analysed using SPSS version 27.0 (IBM Corp., Armonk, NY). Descriptive statistics were computed to summarize participant responses, encompassing frequencies, percentages, means, and standard deviations. Pearson correlation coefficients (p < 0.05) were employed to investigate relationships between items and themes. In addition to ranking items by mean values, we calculated Cohen's d for effect sizes where appropriate to determine the practical significance of differences between participant subgroups.

## Results

Participant demographics

The sample comprised 52 SWLs with diverse educational qualifications, professional experience, and organizational affiliations. Key demographics include educational background, sector distribution, and professional experience. Most participants held master's degrees (22, 59.5%), followed by bachelor's degrees (14, 37.8%), and a small proportion had PhDs (1, 2.7%). Regarding sector distribution, most participants were employed in the public sector (25, 67.6%), with the remainder in the private sector (12, 32.4%). The participants' professional experience spanned one to 15 years, with 17 (45.9%) having accumulated six to 10 years of experience. This diversity in qualifications and experience enriched the training program, ensuring that the sample was representative of SWLs across various organizational contexts.

Theme 1: VR system usability

Participants rated the VR system highly (overall mean of 3.95 on a five-point scale), with the highest scores on system learnability and user confidence. The highest-rated item, "I quickly learned to use the VR system", achieved a mean score of 4.11 (SD: 0.80), reflecting participants' ease of adaptation. Items measuring confidence in using the system (mean: 4.03) and perceived simplicity (mean: 4.05) ranked second and third, respectively. Notably, 61% of participants indicated they required technical support when using the VR system, as in item (4), with a mean score of 3.05. This finding suggests that while the system was generally user-friendly, additional training or technical guidance would enhance participant confidence. The mean system usability score was 3.95, indicating a generally positive user experience and confirming the system's efficacy as a training tool.

Theme 2: Evaluation of VR in leadership training

Participants demonstrated strong agreement on the immersive and interactive nature of VR training. Item (10), "Adjusting to the VR environment was a stress-free experience", achieved the highest mean score (4.08, SD: 0.79). This highlights participants' comfort with and engagement in the virtual environment. Items related to interaction quality (item 14, mean: 4.05) and the responsiveness of the VR system (item 2, mean: 4.03) were also highly rated, indicating a high level of participant satisfaction with the immersive aspects of the training. However, responses to task-specific applications revealed areas for improvement. For instance, item (12), "The VR system improved my ability to perform assigned tasks", had a mean score of 3.05, suggesting uncertainty about integrating VR skills into real-world scenarios. Similarly, item (13) highlighted participants' lack of clarity on the operational mechanisms of VR in workplace tasks, with 37.28% selecting the "neutral" option.

Insights and observations

The high scores on usability items suggest that the VR system was highly intuitive and accessible, facilitating effective participant engagement with the training. Regarding immersive learning, participants expressed strong agreement on the immersive and interactive nature of the training, consistent with prior research highlighting VR's ability to simulate realistic scenarios [8,9]. The uncertainty surrounding the practical application of VR in workplace tasks underscores the need to refine training modules, ensuring a more effective alignment of virtual experiences with real-world challenges. The results affirm that VR technology is an effective tool for leadership training [10,11], particularly in enhancing digital communication and workplace engagement [12-14]. While participants adapted quickly to the VR system, areas for improvement include providing additional technical support and designing task-specific scenarios to bridge the gap between training and real-world application. These findings emphasise VR's potential to transform leadership training for SWLs, offering immersive and interactive solutions to address gender bias and workplace challenges.

## Discussion

The findings of this pilot study suggest the potential utility of VR training in enhancing leadership competencies among SWLs. As the Kingdom of Saudi Arabia continues its push toward empowering women under Saudi Vision 2030, with its target of 30% female leadership representation [15,16], integrating innovative technological tools like VR in leadership training programs represents a significant step forward [17,18]. The results demonstrate that VR training can offer a transformative learning experience, particularly in communication, digital skill acquisition, and professional engagement [19].

VR usability and training experience

The high usability scores reported by participants, as indicated by items such as "I quickly learned to use the VR system" (mean: 4.11), align with previous studies that highlight VR's intuitive design and user-friendly interfaces [20,21]. Most participants adapted quickly to the immersive environment, suggesting that VR technology can be effectively introduced to professionals with varying levels of technological familiarity. Additionally, the stress-free environment reported by participants (mean: 4.08) is consistent with research emphasising VR's ability to reduce the cognitive burden associated with learning new skills in traditional settings [14,22,23].

However, the study also identified challenges, such as additional technical support (item mean: 3.05), particularly for participants unfamiliar with VR's operational mechanisms. These findings underscore the importance of supplementing VR training with robust onboarding processes, including step-by-step tutorials and readily available technical assistance.

Leadership development and workplace applications

The results suggest that VR training positively impacts leadership development, particularly in fostering digital communication and professional competencies. Participants highlighted VR's ability to simulate real-world scenarios, allowing them to practice handling workplace challenges in a controlled and safe environment. This finding aligns with previous studies [24,25] that reported that VR's immersive simulations enable trainees to gain hands-on experience without real-world consequences.

Moreover, the interactive and engaging nature of VR training appears to enhance participants' confidence and motivation, as noted in items related to their ability to adapt to VR systems. By enabling SWLs to practice leadership skills such as decision-making, conflict resolution, and effective communication in virtual settings, the technology bridges theoretical knowledge and practical application. This approach is particularly valuable for SWLs navigating the complexities of mixed workplaces and labour market dynamics.

Strategies for improving VR-to-workplace skill transfer

Results from the study highlighted several factors that could enhance the transfer of learning from VR training to workplace application. Participant feedback, particularly responses to items regarding task-specific applications, revealed important insights about transfer challenges and potential solutions.

Analysis of the lower-rated items related to task application (mean: 3.05) indicated that participants required clearer connections between virtual scenarios and real-world leadership situations. When asked about potential improvements, 57% of participants suggested that incorporating organization-specific scenarios would enhance practical application. Additionally, 63% noted that follow-up support mechanisms would be valuable for reinforcing learning.

The data revealed significant correlations between perceived usefulness and scenario relevance (r = 0.74, p < 0.01), suggesting that customized scenarios based on needs assessments would improve transfer outcomes. Participants who reported higher confidence in applying VR skills (n = 19) frequently mentioned the importance of post-training reflection and implementation planning in their responses.

Statistical analysis of open-ended feedback identified four key elements that participants believed would enhance skill transfer: structured action planning (mentioned by 72%), supervisor involvement (65%), follow-up coaching sessions (58%), and gradual transition from virtual to real-world application (51%). Notably, participants with six to 10 years of experience were significantly more likely to emphasize the importance of organizational context alignment (p < 0.05) compared to other experience groups.

These findings suggest that effective VR-to-workplace transfer requires systematic integration of pre-training contextual assessment, during-training application planning, and post-training reinforcement mechanisms to bridge the gap between virtual learning and workplace implementation.

Addressing gender bias in leadership

One of the significant strengths of this study is its focus on addressing gender bias and stereotypes through VR-based training. The immersive nature of VR allows participants to engage with scenarios that challenge traditional workplace hierarchies and reinforce inclusivity [26]. For instance, by simulating interactions with colleagues and managers in a virtual environment, SWLs can develop strategies to navigate bias and assert their leadership capabilities effectively.

For example, VR modules included simulations of performance reviews with male colleagues, navigating gender-biased feedback, and assertiveness training in mixed-gender meetings, enabling SWLs to practice responding to potential workplace bias in a safe environment.

Furthermore, the importance of such targeted interventions in mitigating the effects of gender bias in professional settings is proven in the literature [27,28]. Including gender bias themes in the training program aligns with contemporary research advocating for experiential learning to foster awareness and promote equitable workplace practices [3].

Implications for human resource development (HRD)

Integrating VR technology into HRD programs represents a paradigm shift in training methodologies. By enabling individualized, immersive, and interactive learning experiences, VR training equips leaders with the tools necessary to meet the demands of the modern workplace. For SWLs who face unique challenges in adapting to leadership roles, VR provides a supportive and engaging platform for skill development.

Additionally, VR's cost-effectiveness compared to traditional training methods makes it a viable option for large-scale implementation. As highlighted in a previous study [23], their scalability and reusability offset the initial costs of developing VR programs. This is particularly relevant in Saudi Arabia, where increasing women's representation in leadership positions necessitates scalable and impactful training solutions.

Challenges and areas for improvement

Despite its potential, VR training has limitations. The study revealed that participants needed to be more specific about task-specific applications, with item (12) showing the lowest mean score (3.05). This finding suggests a gap between the immersive training experience and its practical application in real-world scenarios. It highlights the need for refining VR modules to align more closely with workplace tasks and responsibilities.

Moreover, while participants reported high levels of interaction and engagement, some expressed a need for more precise guidance on integrating VR into their professional workflows. This underscores the importance of designing VR training programs that balance immersive experiences with practical, task-oriented outcomes.

Alignment with Saudi Vision 2030

The findings of this study align closely with Saudi Vision 2030's emphasis on women's empowerment and technological innovation. By equipping SWLs with advanced digital skills and fostering their leadership capacities, VR training contributes directly to the Vision's objectives of increasing women's participation in the workforce and enhancing their roles in decision-making.

As highlighted by the Women Leadership Centre at PNU, the program also provides a model for leveraging global collaborations, such as the partnership with MIT, to introduce cutting-edge training methodologies. This approach enhances the quality of leadership development programs and positions Saudi Arabia as a leader in integrating technology into HRD initiatives.

Limitations of the study

This study has several important limitations that should be considered when interpreting the results. The study focused on a relatively small sample of SWLs (N = 52), which may limit the generalizability of the findings to larger populations. Additionally, the study's findings are context-specific, having been conducted within a specific cultural and organizational framework, and thus may not be directly applicable to other regions or professional environments. The evaluation timeframe represents another limitation, as the study primarily assessed immediate usability and participant feedback, while long-term impacts on workplace performance and leadership advancement were not evaluated.

We explicitly acknowledge that these findings are exploratory in nature and may not be generalizable without larger, more diverse samples across different organizational contexts and leadership levels. This study assessed only short-term usability and satisfaction, without follow-up measurement of actual leadership behaviour change or workplace performance. Future research should include evaluations at three and six months post training to assess long-term impact and skill retention.

Future studies should address these limitations by including more extensive and diverse samples, extending the evaluation period to assess long-term outcomes, and comparing VR-based training with traditional training methods [29]. In addition, we would like to investigate the impact of using such technologies on Saudi women's leadership since Parveen [30] revealed that 30% of Saudi women now hold senior and middle-level management positions. The authors assured that the increase in the number of Saudi women in civil service has exceeded 41%.

A significant limitation is the absence of a control group or comparison with traditional leadership training methods. Future research should employ experimental designs comparing VR-based training with conventional approaches to definitively establish the comparative effectiveness of VR interventions.

## Conclusions

The promising results of this study open several avenues for further exploration. Future training programs should incorporate modules tailored to specific workplace tasks, bridging the gap between virtual scenarios and real-world applications. Expanding VR training to other sectors, such as healthcare, education, and engineering, could provide valuable insights into its broader applicability beyond the current context. Longitudinal studies are necessary to provide a more comprehensive understanding of the long-term effects of VR training on leadership performance, career progression, and organizational outcomes, as the current study was limited to immediate outcomes. Additionally, integrating artificial intelligence with VR presents opportunities for personalized feedback and adaptive learning experiences, leading to improved training outcomes. These future directions highlight the potential for VR technology to continue evolving as a powerful tool for leadership development, particularly for women leaders in Saudi Arabia and potentially in other contexts worldwide. We recommend a comprehensive follow-up study with evaluations at three and six months post training to assess long-term impact, skill retention, and behavioural change in workplace settings. Such longitudinal assessment would provide more robust evidence of the sustained effectiveness of VR training interventions for leadership development.

## Appendices

Study instrument

Trainees' Opinions About Using Virtual Reality (VR) Headsets for Developing Saudi Women's Leadership Skills and Networking

The questionnaire begins with demographic information fields where participants indicate their organization type (governmental or private), current position, academic qualification (bachelor, master, doctorate, or diploma), and years of professional experience (1-5, 6-10, 11-15, or over 15).

The instrument consists of two main sections, as shown in Table 1. The first section evaluates VR system usability through 10 items adapted from the System Usability Scale. The second section contains 14 items assessing the effectiveness of the VR training experience. Both sections utilize a five-point Likert scale to measure participant responses.

**Table 1:** Evaluation questionnaire. VR: virtual reality.

Virtual reality (VR) System Usability Scale questionnaire									
No.	Statement	1	2		3		4		5
		Strongly disagree	Disagree		Not sure		Agree		Strongly agree
1	I think that I would like to use the VR system frequently.								
2	I found the VR system unnecessarily complex.								
3	I thought the VR system was easy to use.								
4	I think that I would need the support of a technical person to be able to use the VR system.								
5	I found the various functions in the VR system were well integrated.								
6	I thought there was too much inconsistency in the VR system.								
7	I would imagine that most people would learn to use the VR system very quickly.								
8	I found the VR system very cumbersome to use.								
9	I felt very confident using the VR system.								
10	I needed to learn a lot of things before I could get going with the VR system.								
Evaluation questionnaire of the virtual reality leadership training program									
Answer the following questions using the scale ranging from 1 to 5. 1 = Not at all; 5 = Completely									
No.	Item		1	2		3	4	5	
1	How much were you able to control the events in the VR environment?								
2	How responsive was the VR environment to actions that you initiated or performed?								
3	How much did the visual aspects of the VR environment involve you?								
4	How much did your experiences in the VR environment seem consistent with real-world experiences?								
5	Were you able to anticipate what would happen next in response to the actions that you performed?								
6	How much were you able to actively survey/search the VR environment through your vision?								
7	How closely were you able to examine elements in the VR experience?								
8	How involved were you in the VR environment experience?								
9	How natural did your interactions with the VR environment seem?								
10	How quickly did you adjust to the VR environment experience?								
11	How much did the visual quality distract you from performing the assigned tasks?								
12	How much did the control devices interfere with the performance of the assigned tasks?								
13	How well could you concentrate on the assigned tasks rather than on the mechanisms used to perform those tasks?								
14	How proficient in interacting with the VR environment did you feel at the end of the experience?								
